# CTLA-4 Expression and Polymorphisms in Lung Tissue of Patients with Diagnosed Non-Small-Cell Lung Cancer

**DOI:** 10.1155/2013/576486

**Published:** 2013-07-09

**Authors:** Adam Antczak, Dorota Pastuszak-Lewandoska, Paweł Górski, Daria Domańska, Monika Migdalska-Sęk, Karolina Czarnecka, Ewa Nawrot, Jacek Kordiak, Ewa Brzeziańska

**Affiliations:** ^1^Department of General and Oncological Pulmonology, 1st Chair of Internal Diseases, Medical University of Lodz, Kopcińskiego 22, 90-153 Łódź, Poland; ^2^Department of Molecular Bases of Medicine, 1st Chair of Internal Diseases, Medical University of Lodz, Pomorska 251, 92-213 Łódź, Poland; ^3^Department of Pneumology and Allergology, 1st Chair of Internal Diseases, Medical University of Lodz, Kopcińskiego 22, 90-153 Łódź, Poland; ^4^Department of Chest Surgery, General and Oncological Surgery University Hospital No. 2, Medical University of Lodz, Żeromskiego 113, 90-710 Łódź, Poland

## Abstract

Cytotoxic T-lymphocyte-associated antigen-4 (CTLA-4) is a potent immunoregulatory molecule that downregulates T-cell activation and thus influences the antitumor immune response. CTLA-4 polymorphisms are associated with various cancers, and CTLA-4 mRNA/protein increased expression is found in several tumor types. However, most of the studies are based on peripheral blood mononuclear cells, and much less is known about the relationship between CTLA-4 expression, especially gene expression, and its polymorphic variants in cancer tissue. In our study we assessed the distribution of CTLA-4 two polymorphisms (+49A/G and −318C/T), using TaqMan probes (rs231775 and rs5742909, resp.), and *CTLA-4* gene expression in real-time PCR assay in non-small-cell lung cancer (NSCLC) tissue samples. The increased *CTLA-4* expression was observed in the majority of NSCLC patients, and it was significantly correlated with TT genotype (−318C/T) and with tumor size (T2 versus T3 + T4). The presence of G allele and GG genotype in cancer tissue (+49A/G) was significantly associated with the increased NSCLC risk. Additionally, we compared genotype distributions in the corresponding tumor and blood samples and found statistically significant differences. The shift from one genotype in the blood to another in the tumor may confirm the complexity of gene functionality in cancer tissue.

## 1. Introduction

The incidence and development of cancer are closely related to dysfunction of immune function. Clinical data indicate an increased risk for tumor development in individuals who are immunesuppressed [1], and, on the other hand, the improved overall and progression-free survival associated with dense intratumoral lymphocyte infiltration of lesions [2]. Human can initiate immune response towards tumors by means of several different mechanisms. Among them the most well documented is cytotoxicity of T cells. As cancer cells have developed multiple mechanisms to evade the immune response [3] proper function of cytotoxic T lymphocytes (CTLs) is critical for immunosurveillance. CTLs after activation are able to recognize and kill autologous cancer cells. The first activating signal is delivered by the tumor-associated antigens (TAAs) displayed by major histocompatibility complex (MHC) class I molecules on antigen-presenting cells (APCs) [4, 5], and it provides specificity to the response. The second, so-called the “costimulatory signal”, as it stimulates T cells in conjunction with antigen, is provided by molecules on APCs that engage particular costimulatory receptors on T cells. The best known is the CD28 receptor, which binds to two costimulatory molecules, B7-1 (CD80) and B7-2 (CD86) [6]. The B7-CD28 interactions stimulate T-cell proliferation, differentiation, and survival.

However, in patients with cancer, the weaken immune response is observed. In this regard cytotoxic T-lymphocyte-associated antigen-4 (CTLA-4) is of significant importance. This molecule is a homodimeric glycoprotein receptor on CTLs and CD28 homologue that also binds to B7-1 and B7-2, expressed after T-cell activation [6]. By inhibiting interleukin-2 production CTLA-4 blocks cell-cycle progression, leading to induction and maintenance of T-cell tolerance. Under physiological conditions, it reduces T-cell response to foreign antigens as well as to autoantigens. In the presence of the tumor's microenvironment, the CTLA-4 molecule is upregulated on the T cells with the help of TGF-beta, a suppressive cytokine secreted by the tumor cells. It has been hypothesized that during the early stage of tumorigenesis, CTLA-4 may elevate the T-cell activation threshold, thereby attenuating the antitumor response and increasing cancer susceptibility [7]. Additionally, the results of several studies suggest that CTLA-4 molecule might be involved in the control of other functions, not only in the inactivation of T-cell response. CTLA-4 mRNA or/and protein expression has been observed on different types of non-T cells, for example, placental fibroblasts, cultured muscle cells, monocytes, and, moreover, neoplastic cells, including leukemic and solid tumor-derived cells [8]. 

The *CTLA-4* gene is located on chromosome 2q33. The function of gene can be influenced by number of genetic variations, including single nucleotide polymorphisms (SNPs), and resulting in disease phenotypes. Indeed, as evidenced in our previous study and in numerous others, several *CTLA-4* polymorphisms that may influence gene expression, lead to amino acid substitution, and alter mRNA splicing have been linked with susceptibility to autoimmune diseases, including autoimmune thyroid diseases, systemic lupus erythematosus, rheumatoid arthritis, and type 1 diabetes [9–15]. Thus, it is hypothesized that in cancer disease antitumor responses such as proliferation and activation of tumor-specific CTLs may also be altered by genetic polymorphisms in *CTLA-4* gene. However, despite the large number of studies of *CTLA-4* SNPs in autoimmune diseases, so far there is still little published data regarding human cancers [16–18]. Although the most recently published meta-analysis proved the association between *CTLA-4* SNPs and the risk of multiple cancer it included only two reports on lung cancer [19].

Lung cancer is a leading cause of cancer-related death worldwide, and the morbidity and the mortality rates are similar, which is a huge clinical problem [20]. The prognosis in lung cancer is poor and limited by the difficulties of diagnosis at early stage of the disease. Therefore, the advance in identification of genetic factors that influence the susceptibility to lung cancer, especially to non-small-cell lung cancer (NSCLC), which is the most frequent lung cancer type, is of great value.

The aim of our study was to evaluate the level of *CTLA-4* mRNA expression in lung cancer tissue and to assess its relationship with two chosen *CTLA-4* polymorphisms, affecting the leader sequence of CTLA-4 protein (+49A/G) and the promoter (−318C/T) of the gene. Additionally, we were interested in the question of whether the distribution of *CTLA-4* genotypes was the same in the blood and the corresponding cancer tissue. We also focused on the associations between *CTLA-4* expression and gene polymorphic variants and NSCLC patients characteristics, as well as tumor clinical staging.

## 2. Material and Methods

The study has been approved by the Ethical Committee of the Medical University of Lodz, Poland. 

### 2.1. Patients Characteristics

Seventy-one patients with diagnosed NSCLC (25 women, mean age 63 ± 8.717, range 47–79 and 46 men, mean age 65 ± 8.234, range 47–87) have been enrolled into the study. They underwent lobectomy at the Department of Chest Surgery, General and Oncological Surgery University Hospital No. 2, Medical University of Lodz. During the surgery, tumor tissue samples (100–150 mg) were obtained, collected in lysis buffer (Buffer RNAlater, Qiagen Sciences, USA), and frozen at −70°C until use. 

According to the pathomorphological reports, the study material included adenocarcinoma (AC, *n* = 23), squamous cell carcinoma (SCC, *n* = 41), and large cell carcinoma (LCC, *n* = 7).  Table 1 shows the studied tumor samples characteristics according to TNM (tumor, node, and metastasis) and AJCC (American Joint Committee on Cancer) staging systems.

Regarding the smoking history, 90% of the NSCLC patients (*n* = 64) were smokers. They were divided into two groups: one with smoking level ≤30 pack-years (*n* = 26) and the other with smoking level >30 pack-years (*n* = 38).

Control group consisted of 104 healthy persons without lung cancer in the history. Peripheral blood samples (5 mL) were collected on EDTA and frozen at −70°C until use.

### 2.2. Relative Expression

Total RNA was isolated from biological material (cancer tissue obtained from the center of lung lesion and macroscopically unchanged lung tissue obtained from the most distant site from the resected lesion), using Universal RNA Purification Kit (Eurix, Poland), according to the manufacturer's procedure. The quality and quantity assessments of total RNA samples were determined by minielectrophoreses in polyacrylamide gel (Agilent 2100 Bioanalyzer, Agilent, USA), using RNA 6000 Pico/Nano LabChip kit (Agilent Technologies, USA). Reverse transcription reaction was performed using 100 ng of total RNA, in a total volume of 20 *μ*L, using High-Capacity cDNA Reverse Transcription Kit (Applied Biosystems, USA), in the following conditions: 10 minutes at 25°C, followed by 120 minutes at 37°C; then the samples were heated to 85°C for 5 seconds and hold at 4°C. Gene relative expression was assessed using TaqMan probes for the studied *CTLA-4* gene and the reference gene *β*-actin (Hs00175480_m1 and Hs99999903_m1, resp.; TaqMan Gene Expression Assays, Applied Biosystems) in 7900HT Fast Real-Time PCR System (Applied Biosystems, USA). PCR reactions were carried out for 40 cycles with annealing temperature of 60°C. The reaction was repeated 3 times. The expression level (RQ value) was calculated based on the delta-delta *C*_*T*_ method adjusted to *β*-actin expression, for each sample. Macroscopically unchanged lung tissue served as calibrator sample. 

### 2.3. SNP Genotyping

Total genomic DNA was isolated from lung tissue and peripheral blood samples of NSCLC patients as well as peripheral blood samples of healthy controls using Qiagen QIAamp DNA Mini Kit (Qiagen Sciences, USA), according to the manufacturer's protocol. *CTLA-4* gene polymorphisms were examined using TaqMan Universal PCR Master Mix, DNA (20 ng/*μ*L) and TaqMan probes (rs231775 and rs5742909) in 7900HT Fast Real-Time PCR System using TaqMan 5′ allelic discrimination assay (Applied Biosystems, USA).

### 2.4. Statistical Analysis

For statistical analysis, STATA version 10 (State College, TX, USA) was used. Compliance with Hardy-Weinberg equilibrium (HWE) was assessed using the chi-square test (*χ* ^2^) and Fisher's test. Odd ratios (OR) and its 95% confidence intervals (95% CI) were calculated as an assessment of the strength and direction of association between the studied individual genotypes and susceptibility to NSCLC. Kruskal-Wallis or Mann-Whitney *U* tests were used to assess the associations between gene polymorphic variants as well as expression levels and NSCLC histological subtypes, tumor staging (TNM and AJCC systems), patient age, gender, and smoking history. To compare allele/genotype distributions between the tumor tissue and blood samples *χ* ^2^ test was used. Linkage disequilibrium (LD) in the two studied polymorphic sites was evaluated using Fisher's exact test. In all tests *P* value below 0.05 (*P* < 0.05) was considered as statistically significant.

## 3. Results

### 3.1. *CTLA-4 *Expression

The obtained RQ values, calculated based on the delta-delta *C*_*T*_ method, adjusted to the *β*-actin expression, and relative to the expression level of calibrator (for which RQ = 1), were compared among NSCLC patients in regard to histopathological NSCLC subtypes, tumor staging (TNM, AJCC), patients' age, gender, and smoking history, as well as to *CTLA-4* polymorphic variants. Generally, *CTLA-4* expression was increased in the majority of NSCLC patients (74.65%), with the mean RQ value of 6.91.  Table 2 specifies the results of relative expression analysis, indicating mean RQ values, as well as number of samples with the increased (RQ value >1) and decreased (RQ value <1) *CTLA-4* expression in comparison with calibrator.

Statistical analysis revealed significant association between the increased gene expression level and tumor staging according to TNM staging: in T2 tumors *CTLA-4 *expression was significantly higher than in T3 + T4 group (*P* = 0.007, Newman-Keuls test). There were no statistically significant differences in gene expression in T1 tumors versus T2 tumors (*P* = 0.12) and versus T3 + T4 group (*P* = 0.50). Regarding the studied polymorphisms, the significantly increased expression of *CTLA-4* was found for *CTLA-4* −318C/T SNP, namely, for TT genotype as compared with CC genotype (*P* = 0.008, Newman-Keuls test). There were no statistically significant associations between other −318C/T genotypes (CT versus TT, *P* = 0.84; CT versus CC, *P* = 0.80) and between +49A/G genotypes and *CTLA-4* expression levels (*P* = 0.53). 

There was no statistically significant differences (*P* > 0.05) for the other variables studied, that is, histopathological subtypes (SCC versus AC versus LCC; SCC versus NSCC), AJCC staging, patients' age, gender, and the history of smoking.

### 3.2. *CTLA-4* +49A/G and −318C/T Polymorphisms

#### 3.2.1. NSCLC Tissue

The studied polymorphisms were in Hardy-Weinberg equilibrium both in the NSCLC patients and in the controls.

Statistical analysis confirmed the presence of significant differences between the studied groups (NSCLC versus control) concerning the distribution of *CTLA-4* +49A/G genotypes (AA, AG, and GG) and the individual alleles. The obtained results summarized in Table 3 indicate that the presence of G allele and GG genotype may lead to the increased NSCLC risk, while the presence of A allele and AA genotype may reduce the risk of lung cancer development.

 Regarding −318C/T polymorphism, there were no statistically significant differences in genotypic or allelic distributions between NSCLC patients and the control group (see Table 3). 

 We did not reveal any statistically significant associations (*P* > 0.05) between the studied *CTLA-4* polymorphisms and patients' gender, age, smoking history, individual NSCLC subtypes, TNM, and AJCC classification.

#### 3.2.2. Peripheral Blood Mononuclear Cells

The studied polymorphisms were in Hardy-Weinberg equilibrium both in the NSCLC patients and in the controls. The results of statistical analysis concerning the distribution of the studied genotypes in PBMCs are summarized in Table 4.

 No statistical significances were found (*P* > 0.05) regarding the association between the studied *CTLA-4* polymorphisms in PBMCs and patients' characteristics (age, gender, smoking history), NSCLC subtypes (SCC, AC, and LCC) and tumor staging (TNM and AJCC systems).

### 3.3. *CTLA-4* Genotype Distributions in NSCLC Tissue versus Blood Samples

 Comparing the results of *CTLA-4* SNP genotyping in the tumor tissue and the corresponding blood sample, a shift from one genotype to another in more than 60% of patients was observed, for both studied *CTLA-4* polymorphisms. Statistically significant difference was found in case of +49 *CTLA-4* SNP (*P* = 0.01, *χ* ^2^ test) when comparing the samples with and without genotype shift, while in case of −318 *CTLA-4* SNP it did not reach the statistical significance (*P* = 0.08, *χ* ^2^ test). Regarding the individual genotypes, statistically significant differences were found for +49 AA → AG shift (*P* = 0.011, *χ* ^2^ test) and −318 CC→CT shift (*P* = 0.017, *χ* ^2^ test).  Table 5 summarizes the differences between the distribution of the studied genotypes in blood and paired tissues in NSCLC patients. 

### 3.4. Linkage Disequilibrium Analysis

 No linkage disequilibrium (LD) was found between the two studied polymorphic sites (*P* > 0.05, Fisher's exact test). 

## 4. Discussion 

 It is widely accepted that *CTLA-4* plays a crucial role in the maintenance of the immune response by its expression on activated and regulatory T cells. Accordingly, it is speculated that extraordinary *CTLA-4* expression could be associated with enhanced susceptibility to tumor growth and/or progression, attenuating the antitumor immune response. And although the significance of CTLA-4 molecule in antitumor immunity has been well recognized, proving its role in T-cell down regulation [21, 22], its expression in a non-immunogenic context has not been well studied. 

### 4.1. CTLA-4 Expression in Cancer Tissue

There are only a few publications that focused on this subject, and they confirm the expression of *CTLA-4* mRNA and/or protein on tumor cells [23–25] and tumor-derived cell lines, although Contardi et al. [8] found no correlation with tissue origin. Recently, CTLA-4 protein expression has been reported to be significantly higher in primary lung cancer samples and precancerous lesions than in normal lung tissue [24]. 

The aim of our work was to assess the level of *CTLA-4* mRNA expression in lung cancer tissue, and we confirmed the increased gene expression in nearly 75% of NSCLC samples. To our knowledge, it is the first such study performed on mRNA level using real-time PCR method. The other available studies performed in lung cancer cells focused on CTLA-4 protein expression [24, 26, 27]. Among them, the report of Salvi et al. [26] was the first one suggesting a prognostic role of CTLA-4 expression in NSCLC.

In our analysis we observed statistically significant association among the increased *CTLA-4* mRNA expression and tumor size. It was significantly higher in T2 group as compared to the group combining T3 and T4 samples. Also, the findings of others [26] indicate a tendency of favourable independent influence of CTLA-4 protein overexpression on patients' overall survival with better prognosis in those with higher CTLA-4 expression. The study of Contardi et al. [8] undoubtedly indicated that CTLA-4 is able to specifically transduce an apoptotic signals into tumor cells, comparable with the ones observed in T cells. In this way, CTLA-4 molecule as a negative regulator of tumor cell proliferation might influence the clinical outcome of patients with cancer. Our results indicating *CTLA-4* overexpression in T2 versus T3 and T4 tumors could support in a sense this hypothesis. 

The levels of *CTLA-4* expression in our study did not differ significantly between the individual NSCLC histopathological subtypes. Similar results, although on protein level, were obtained by Zheng et al. [24], but other study revealed higher CTLA-4 immunoexpression level in nonsquamous as compared to squamous histological type of NSCLC [26]. In our study, we did not observe any differences in *CTLA-4* mRNA expression in NSCC versus SCC.

 We also did not find any associations between *CTLA-4* mRNA expression and patients' age and gender. Generally, it is in accordance with the results of others [24, 26] with the exception for patients' age that was closely related with the positive CTLA-4 protein expression in the study of Zheng et al. [24]. 

Summarizing the previous considerations, it should be emphasized that it is difficult to compare the results of CTLA-4 expression obtained on mRNA and protein level, having regard to the posttranscriptional or posttranslational modifications that could take place.

### 4.2. Functional Significance of *CTLA-4* Polymorphisms

The altered expression of *CTLA-4* may be influenced by genetic variations in the gene, including single nucleotide polymorphism. There are numerous reports assessing the association between *CTLA-4* genotypes and cancer risk: +49A/G SNP has been found in breast, esophageal, gastric and colorectal cancers, oral squamous cell carcinoma, and osteosarcoma [17, 27–31], while −318C/T SNP, although less frequently analyzed, has also revealed associations with breast, cervical, gastric and colorectal cancers, and B-cell chronic lymphocytic leukemia [29, 32–35]. Polymorphisms in *CTLA-4* are regarded as genetic susceptibility factors; however, there are reports that could not establish any associations between different *CTLA-4* SNPs and several types of cancers [29, 36]. 

#### 4.2.1. CTLA-4 +49A/G Polymorphism

The available data analyzing the functionality of *CTLA-4* SNPs are limited. Polymorphism at position +49A/G is associated with adenine (A) to guanine (G) transition within exon 1 of *CTLA-4* gene coding the leader sequence of the CTLA-4 protein, and resulting in (17)Thr to (17)Ala amino acid substitution. This functionally affects T-cell activation: CTLA-4-(17)Ala homozygotes (+49GG genotype) express one-third less CTLA-4 protein on the surface of their T cells than CTLA-4-(17)Thr homozygotes (+49AA genotype) [37], thus exerting less profound inhibitory effect on T-cell proliferation [38]. This could be associated with better antitumor immune response and decreased cancer risk. In our study group only two patients had +49GG genotype in PBMCs, and it is too small to regard the results of OR analysis as reliable. On the other hand, we did not observe any significant association between +49AA genotypes—constituting the majority in the studied group—and increased NSCLC risk. Another study conducted in Polish population revealed a trend toward overrepresentation of A allele carriers (AA, AG) among patients as compared with controls, however, the statistical significance was reached only in women [16]. The studies performed in Asian population established +49AA genotype as an independent adverse prognostic factor in NSCLC patients [27, 39]. However, population-based differences cannot be excluded.

In our study, lack of association between +49 *CTLA-4* SNP and gene expression may indicate the significance of posttranscriptional or posttranslational modifications, with similar amounts of starting mRNA irrespective of the genotype. The results of several studies indicate that the polymorphism in *CTLA-4* exon 1 may influence the rates of endocytosis or surface trafficking, glycosylation of CTLA-4 or lower capability to bind B7.1 [27, 37, 38], resulting in lower CTLA-4 expression on the surface of T cells and thereby forming the mechanism responsible for weaker inhibitory effect of GG genotype on T-cell activation.

Interestingly, a genotype shift observed in our study between the blood and the corresponding tumor samples revealed the presence of G allele in the majority of tissue samples (73.24%). The differences regarding genotype distribution in PBMCs and tumor specimens were statistically significant. Indeed, there are reports describing a shift from one genotype in the blood to another genotype in the tissue and proving its role as a prognostic factor, as in ovarian cancer patients [40]. To our knowledge, the result of our study revealing the genotype shift in blood versus NSCLC tissue is the first one. Moreover, the genotypes found in our tumor samples were significantly associated with NSCLC risk. 

#### 4.2.2. CTLA-4 −318C/T Polymorphism

The other studied polymorphism, −318C/T, is associated with cytosine- (C-) thymine (T) single-base substitution within the promoter sequence and is likely to affect the gene expression. Indeed, it has been reported that the presence of −318T allele is associated with higher promoter activity [41–43] and significantly increased expression of mRNA and surface CTLA-4 [44], enabling more potent inhibition of T-cells activation. This may eventually lead to the decreased antitumor immunity and to cancer susceptibility. 

In fact, our analysis of *CTLA-4* expression revealed the significantly higher mRNA expression level in the lung cancer tissue samples carrying −318TT genotype. This confirms the functionality of the T allele substitution in the place of the C allele in the promoter of* CTLA-4*. 

In our study we could not establish any significant association between −318C/T SNP and NSCLC risk, neither in tumor tissue nor in blood. However, in another study involving Polish patients, statistical significance for T allele and T-allele carrying genotypes (TT and CT) was obtained [16], although observed only for NSCLC female patients. The results of meta-analysis involving several types of cancer and including lung cancer [19] revealed a significantly increased risk of cancer in Europeans carrying T allele (TT and TC genotypes) but not in Asians or Americans. Indeed, the studies performed in Asian populations found no association between polymorphic variants in the promoter of *CTLA-4* gene and NSCLC [27, 45].

Considering the association between the studied *CTLA-4* polymorphisms and clinicopathological features of NSCLC, including histopathological type of cancer, tumor stage, and patients' gender, we did not observe any significant differences. Similar findings were obtained by others [39, 45] with the exception of one study [16] regarding patients' gender (women) and +49 *CTLA-4* SNP. 

## 5. Conclusions

The presented study carried out in NSCLC tissue and blood samples, combining the CTLA-4 expression analysis on mRNA level with gene polymorphic variants, seems valuable as it expands a small amount of data available in lung cancer patients. It should be noted that till now only four studies investigating *CTLA-4 *polymorphisms in NSCLC have been published [16, 27, 38, 39]. In our study we showed allelic and genotypic distribution of +49 and −318 *CTLA-4* SNPs in a group of NSCLC patients of Caucasian origin and revealed *CTLA-4* genotype distribution shift from one genotype in the blood to another genotype in the tumor. This phenomenon may reflect the complexity of gene functionality in cancer tissue. Our results, indicating the correlation between the C/T substitution in the promoter of the gene and its increased expression, and the observed significant association between the gene expression and the tumor size might provide additional evidence confirming the proapoptotic effect of CTLA-4 on tumor cells. Additionally, the described association between GG genotype in tumor tissue and the increased risk of NSCLC development might confirm the weaker inhibitory effect of this genotype on cell proliferation. 

Although the obtained results are promising, more investigations in the future in a larger group of NSCLC patients are needed. Particularly, if further research—focusing on the association between CTLA-4 SNPs and altered gene expression—could definitively confirm its role as a negative regulator of tumor cell proliferation.

## Figures and Tables

**Table 1:** Tumor characteristics.

Cancer staging system	*n* (%)	SCC *n* = 41 (58%)	NSCC	
			AC *n* = 23 (32%)	LCC *n* = 7 (10%)
TNM				
T1	19 (26.76%)	8	11	0
T2	33 (46.48%)	18	11	4
T3-T4	19 (26.76%)	15	1	3
AJCC				
IA	13 (18.31%)	7	6	0
IB	14 (19.72%)	4	9	1
IIA	16 (22.53%)	12	3	1
IIB	8 (11.27%)	6	0	2
IIIA/IIIB	20 (28.17%)	12	5	3

AC: adenocarcinoma; LCC: large cell carcinoma; NSCC: nonsquamous cell carcinoma; SCC: squamous cell carcinoma.

**Table 2:** RQ values, reflecting the relative expression levels of *CTLA-4*, in NSCLC subtypes and according to the tumor size.

Histopathological NSCLC subtype	Mean RQ value ± SEM	RQ value >1 *n* (%)	RQ value <1 *n* (%)
SCC (*n* = 41)	8.34 ± 16.86	32 (78%)	9 (22%)
AC (*n* = 23)	5.47 ± 9.92	15 (65.22%)	8 (34.78%)
LCC (*n* = 7)	6.93 ± 1.23	6 (85.71%)	1 (14.29%)
Tumor size (TNM system)			
T1 (*n* = 19)	8.29 ± 7.56	13 (68%)	6 (32%)
T2 (*n* = 33)	14.16 ± 18.24	24 (73%)	9 (27%)
T3 + T4 (*n* = 19)	2.13 ± 14.37	11 (58%)	8 (42%)

Total (*n* = 71)	6.91 ± 17.79	53 (74.65%)	18 (25.35%)

**Table 3:** *CTLA-4* +49 A/G and −318 C/T genotype and allele distribution in tumor tissue of NSCLC patients in comparison with the control group.

*CTLA-4* genotypes and alleles	NSCLC patients *n* = 71		Healthy controls *n* = 104		OR (CI 95%)	*P* value
	Number	Frequency	Number	Frequency		
+49 AA	19	0.27	49	0.47	0.41 (0.21–0.78)	0.02
+49 AG	25	0.35	33	0.32	1.16 (0.62–2.21)	0.74
+49 GG	27	0.38	22	0.21	2.23 (1.17–4.48)	0.015

	*χ* ^2^ = 5.83 *P* = 0.63		*χ* ^2^ = 2.41 *P* = 0.83			

+49 A allele	63	0.44	131	0.63	0.47 (0.30–0.72)	0.028
+49 G allele	79	0.56	77	0.37	2.13 (1.38–3.29)	0.028
−318 CC	24	0.33	36	0.34	0.96 (0.91–1.82)	0.15
−318 CT	19	0.27	37	0.36	0.66 (0.34–1.28)	0.97
−318 TT	28	0.40	31	0.30	1.53 (0.81–2.89)	0.41

	*χ* ^2^ = 3.42 *P* = 0.37		*χ* ^2^ = 8.55 *P* = 0.42			

−318 C allele	67	0.47	109	0.60	0.81 (0.53–1.24)	0.32
−318 T allele	75	0.53	99	0.40	1.23 (1.80–1.89)	0.32

**Table 4:** *CTLA-4* +49 A/G and −318 C/T genotype and allele distribution in blood samples of NSCLC patients in comparison with the control group.

*CTLA-4* genotypes and alleles	NSCLC patients *n* = 71		Healthy controls *n* = 104		OR (CI 95%)	*P* value
	Number	Frequency	Number	Frequency		
+49 AA	56	0.80	49	0.47	4.19 (2.11–8.33)	0.58
+49 AG	13	0.18	33	0.32	0.48 (0.23–1.02)	0.23
+49 GG	2	0.02	22	0.21	0.11 (0.02–0.48)	0.03

	*χ* ^2^ = 1.23 *P* = 0.73		*χ* ^2^ = 3.26 *P* = 0.37			

+49 A allele	63	0.88	131	0.63	4.32 (2.42–7.72)	0.47
+49 G allele	79	0.12	77	0.37	0.23 (0.13–0.41)	0.47
−318 CC	59	0.83	36	0.34	9.29 (0.42–9.48)	0.76
−318 CT	10	0.14	37	0.36	0.30 (0.14–2.65)	0.57
−318 TT	2	0.03	31	0.30	0.10 (0.02–0.30)	0.02

	*χ* ^2^ = 2.67 *P* = 0.25		*χ* ^2^ = 4.65 *P* = 0.35			

−318 C allele	128	0.90	109	0.60	8.30 (0.5–15.36)	0.23
−318 T allele	14	0.10	99	0.40	0.12 (0.65–2.22)	0.23

**Table 5:** *CTLA-4* SNP shift between blood and tumor tissue.

+49 *CTLA-4* SNP genotype shift (*n* = 47; 72.31%)			
Genotype shift	AA→AG	AG→GG	GG→AA
No. of patients (%)	24 (51.06)	13 (27.66)	10 (21.28)

−318 *CTLA-4* SNP genotype shift (*n* = 41, 63.10%)			

Genotype shift	CC→CT	CT→TT	CC→TT
No. of patients (%)	17 (41.46)	10 (24.39)	14 (34.15)
